# Neuroworsening in traumatic brain injury: A consensus of the Latin American Brain Injury Consortium (LABIC) and the Latin American Federation of Neurosurgical Societies (FLANC) expert group

**DOI:** 10.1007/s10143-026-04284-z

**Published:** 2026-05-07

**Authors:** Daniel A. Godoy, Robson Luís Oliveira de Amorim, Jorge Luís Paranhos, Kelia Santiago, Wellingson Paiva, Jorge Carrizosa, Franly Vázquez, Piedad Acurio, Jorge Flecha, Pedro Grille, Gustavo Domeniconi, Carlos Romero Patiño, Miguel Ángel Vences, Maximiliano Rovegno, Denise Efren Hermosa Altez, Manuel Jibaja, Rodrigo Faleiro, Marcelo Zenteno, Laura Bottani, Alejandro Rabinstein, Andrés M. Rubiano

**Affiliations:** 1 Meditech Foundation, Bogota, Colombia; 2 Hospital Universitário Getúlio Vargas, Universidade Federal do Amazonas, Manaos, Brazil; 3 Hospital Santa Casa de Sao Joao Do Rei, São João, Minas Gerais Brazil; 4 Hospital João XXIII Belo Horizonte, Belo Horizonte, Minas Gerais Brazil; 5 Divisão de Neurocirurgia, Hospital da Clínicas da Faculdade de Medicina da Universidade de São Paulo, São Paulo, Brazil; 6 Department of Critical and Intensive Care Medicine, Neurointensive Care Unit, Fundación Santa Fe, Bogotá, Colombia; 7 Hospital Salvador B. Gautier. Servicio de Neurocirugía, Santo Domingo, República Dominicana; 8 Departamento de Neurocirugía. Hospital Solca, Tungurahua, Pontificia Universidad Catolica, Quito, Ecuador; 9 Critical Care Medicine. Hospital del Trauma y Hospital Central I.P.S, Asuncion, Paraguay; 10 Unidad de Cuidados Intensivos, Hospital Maciel, ASSE, Montevideo, Uruguay; 11 Unidad de Cuidados Intensivos. Sanatorio de la Trinidad. San Isidro, Buenos Aires, Argentina; 12 Departamento de Medicina Interna, Unidad de Pacientes Críticos. Hospital Clínico Universidad de Chile, Santiago, Chile; 13 Servicio de Neurología, Hospital Nacional Alberto Sabogal Sologuren, Bellavista District, Peru; 14 Departamento de Medicina Intensiva, Facultad de Medicina, Pontificia Universidad Católica de Chile, Santiago, Chile; 15 Division of Neurosciences Critical Care, Department of Neurology and Anesthesiology and Critical Care Medicine, Johns Hopkins University School of Medicine, Baltimore, MD USA; 16 Servicio de Neurocirugía Hospital Nacional Daniel Alcides Carrión, Callao, Perú; 17 Escuela de Medicina, Unidad de Cuidados Intensivos. Hospital Eugenio Espejo, Universidad San Francisco, Quito, Ecuador; 18 Neurosurgery Department, Hospital João XXIII. Hospital Felício Rocho. Faculdade de Ciências Médicas de Minas Gerais, Belo Horizonte, Brazil; 19 Departamento de Neurocirugia, Hospital San Juan de Dios, Tarija, Bolivia; 20 Departamento de Neurocirugia, Hospital eugenio Espejo, Quito, Ecuador; 21 Neurosciences Intensive Care Unit, Mayo Clinic, Rochester, MN USA; 22 Medical and Research Director, MEDITECH Foundation, Calle 7A # 44-103, Cali, Valle del Cauca 760036 Colombia; 23 Professor of Neurosciences and Neurosurgery, Universidad El Bosque, Bogotá, Colombia

**Keywords:** Neuroworsening, Neurologic deterioration, Neurologic impairment, Clinical deterioration, Neuromonitoring, Traumatic brain injury

## Abstract

Neuroworsening (NW) after traumatic brain injury (TBI) is a life-threatening complication affecting at least one in five patients. The current definition remains heterogeneous and does not integrate contemporary neuromonitoring tools that could help reduce this variability. Current diagnostic approaches are predominantly reactive, identifying deterioration only after brain herniation has occurred. To establish an expert consensus to update the definition of NW in TBI by proposing a stratified diagnostic framework aligned with precision and personalized medicine principles, aiming to shift a paradigm that has been in use for the past 50 years. A formal Delphi consensus process was conducted involving 25 experts from the Latin American Brain Injury Consortium (LABIC) and the Latin American Federation of Neurosurgical Societies (FLANC). A pre-consensus systematic literature review was performed, followed by structured electronic surveys with Likert-scale and multiple-choice items. Consensus was predefined as ≥80% agreement for establishing a statement. A 95.2% response rate was achieved in the first Delphi round, with 100% of statements reaching the consensus threshold. The panel agreed on a stratified three phenotype NW framework: Established NW, Subclinical NW and High-Risk Phenotype, including patients with pre-existing anatomical or systemic conditions that affect cerebral compliance or oxygenation reserve. This consensus proposes integrating clinical, imaging, and multimodal neuromonitoring parameters to update the definition of NW, thereby reducing heterogeneity in the current concept. Seven statements were established with >80% agreement. The new definition promotes a preventive approach to this critical condition, in contrast to the traditional reactive model.

**Supplementary Information:**

The online version contains supplementary material available at 10.1007/s10143-026-04284-z.

## Introduction

Neuroworsening (NW) is a clinical, imaging, or monitoring condition that can be identified in acute brain injuries and is associated with serious complications with a significant negative impact on outcomes [1–3]. Its prevalence is high, especially in traumatic brain injury (TBI), present in at least one of every five cases [4]. It can occur at any point in the clinical course, depending on several factors, including the type of associated brain edema and the early access to aggressive surgical therapy if required [5–9]. Its causes are multiple and varied, while its predictors or risk factors remain unclear [4–6]. The confirmation of NW requires an early, multidisciplinary, and intensive approach for diagnosis and management to minimize its consequences. Unfortunately, its definition and the criteria used for this purpose are not universally validated, are different, heterogeneous, and have limitations that should not be minimized [4]. Currently, there is a mismatch between a traditional approach (identification in a delayed stage, with a brain herniation syndrome in process) [10–12] versus the current context of personalized and precision medicine (where emergent neuromonitoring techniques allow early identification of a mismatch between compliance and distensibility inside the skull before a herniation process occurs).

Neglecting the integration of neuroimaging and neuromonitoring (invasive or non-invasive) in the emergency department (ED) and the intensive care unit (ICU) to identify NW as early as possible is a critical mistake in the precision and personalized medicine approach. Evidence of this issue is the absence of updated literature on this specific point of view [4]. No high-level clinical studies test these new perspectives [4]. The traditional way of identifying NW urgently needs a paradigm shift from a reactive to a preventive approach. Today, we are still treating patients in a very delayed fashion, as all the current triggers to start or escalate therapy according to the guidelines, consensus, and protocols are based on late identification, associated with clinical deterioration or neuroimaging findings of brain tissue herniation [10–12].

To address this gap, the Latin American Brain Injury Consortium (LABIC) and the Latin American Federation of Neurological Surgeons (FLANC) Neurotrauma and Intensive Care Chapter developed an expert consensus proposing an updated definition with novel criteria to define NW in the era of precision and personalized medicine. At the same time, a pathway for identifying and guiding management of NW in the context of TBI has been developed.

## Materials and methods

A formal expert consensus Delphi process was established to update and standardize the concept of neuroworsening in traumatic brain injury (TBI) [13]. The total number of experts invited was 25. Of these, only one withdrew from the consensus due to illness. The final composition consisted of 20 individuals who completed the voting process and 4 additional members of the methodological group that does not vote. The members of the Latin American Federation of Neurosurgical Societies (FLANC) and the members of the Latin American Brain Injury Consortium (LABIC), were selected based in specific inclusion criteria: (a) > 10 years’ experience in the management of TBI; (b) active involvement in acute care management of TBI population; (c) representation of pertinent disciplines (neurosurgeons, neurocritical care and general intensivists, neurologists and anesthesiologists); (d) geographic diversity around Latin America (Central, South and Caribbean region); (e) training in systematic searches; and (f) ability to commit time to the statement development process. The working group was divided into two subgroups: one in charge of the design of the exercise, planning it, organizing the logistics and the developing the first document draft (methodological group) who did not participate in the voting process (*n* = 4) and the group of panelists (inclusion criteria; *n* = 20) in charge of the statement developing and voting with active participation in the discussions (expert group). Prior to the consensus, a systematic search in the topic was performed. The methodological group summarized the evidence and shared it with each member of the voting group. Specific details over the voting rounds, and the search strategy are attached as supplementary material. An electronic survey was developed to gather expert opinions on clinical, radiological, and neuromonitoring variables associated with neuroworsening. The poll was constructed using the Google Forms platform (Google LLC, Mountain View, CA, USA) to facilitate asynchronous participation and ensure data integrity. The survey was structured into three sections, including Likert-scale questions for agreement levels, multiple-choice items for clinical parameters, and open-ended fields for qualitative input. The instrument was piloted by a core steering committee to ensure clarity and relevance before final distribution. The digital poll was distributed via email and professional mobile messaging platforms to the selected members. Participants were provided with a standardized set of instructions and a clear definition of the exercise objectives. To minimize bias, responses were collected anonymously over a period of one week. Reminders were sent periodically to ensure a 100% response rate among the invited experts. All raw data were automatically captured into a linked spreadsheet for subsequent quantitative and qualitative analysis. Responses were analyzed to determine the degree of agreement for each statement. Consensus was predefined as an 80% agreement rate among respondents. For items where consensus was not achieved in the initial poll, the results were synthesized and redistributed for further deliberation during the Delphi exercise [13]. Descriptive statistics were used to summarize the panel´s demographic characteristics and the distribution of responses for each clinical variable.

## Results

The first round of the Delphi consensus process achieved a 95.2% response rate, with 20 of 21 invited experts completing the survey. The first Delphi round demonstrated exceptional consensus across all proposed statements, with 100% of statements achieving the predefined consensus threshold of ≥ 80% agreement. More details are shown in the supplemental material.

### Position statement

**Fig. 1:**
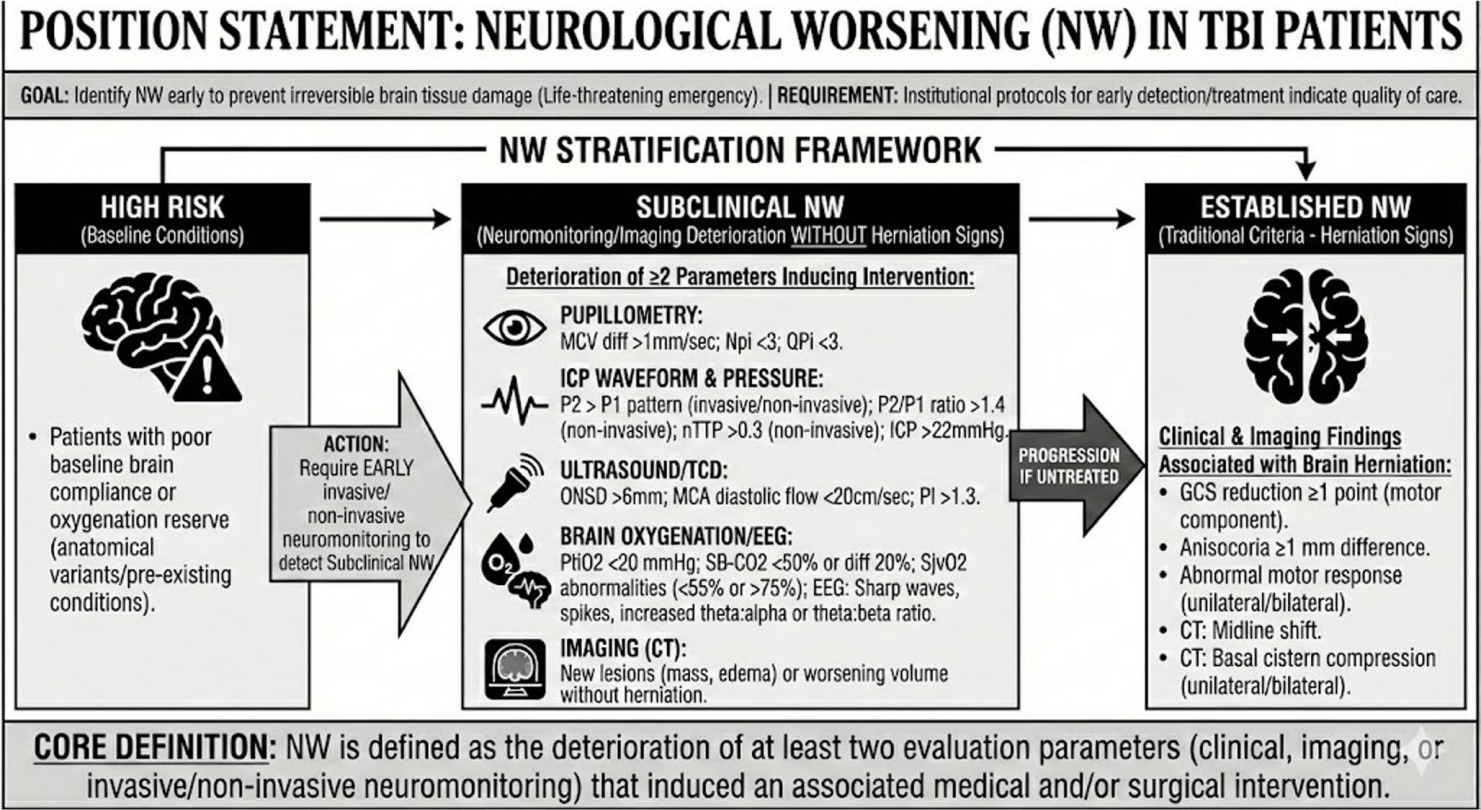
Proposed stratification model to expand the NW definition. NW: neuroworsening; GCS: Glasgow coma scale; CT: computed tomography; NPI: Neurologic pupillary index; QPi: Quantitative Pupillary Index, ICP: intracranial pressure; PbtiO2: parenchymal brain oxygen pressure; SvJO2: venous jugular oxygen saturation; NIRS: near infrared oxygen saturation; EEG: electroencephalography
e *Established NW is defined by traditional criteria (clinical and imaging findings associated with brain herniation)*,* including*:
  *Glasgow Coma Score (GCS) reduction of at least 1 point in the motor component.*
  *Anisocoria of at least 1 mm of difference.*
  *Abnormal motor response (unilaterally or bilaterally).*
  *Midline shift on computed tomography (CT) imaging.*
  *Basal cistern compression (unilaterally or bilaterally) on CT imaging.*
f *Subclinical NW is defined as the deterioration of at least two parameters in neuromonitoring (invasive or non-invasive)*,* or imaging (without herniation signs)*,* inducing medical and/or surgical interventions. These parameters include*:
  *Pupillometry maximum contraction velocity (MCV) difference >1 mm/sec between eyes.*
  *Neurologic pupillary index (Npi) <3.*
  *Quantitative pupillary index (QPi) <3.*
  *Optic nerve sheath diameter (ONSD) >6mm on any side evaluated by ultrasound.*
  *Peak 2 (P2) >Peak 1 (P1) pattern on any side of the skull during invasive or non invasive intracranial pressure (ICP) waveform analysis.*
  *P2/P1 ratio analysis on non-invasive ICP monitoring > 1.4 on any side of the skull.*
  *Normalized time to peak (nTTP) > 0.3 on any side of the skull during non invasive ICP monitoring.*
  *ICP >22mmHg.*
  *Medial cerebral artery (MCA) diastolic flow velocity < 20cm/sec on any side during transcranial Doppler (TCD) monitoring.*
  *Pulsatility index (PI) > 1.3 on any side during TCD monitoring.*
  *Sharp waves, spikes, or spike-and-wave complexes or increased theta: alpha ratio or theta: beta ratio in any area during EEG monitoring.*
  *Brain oxygen tissue pressure (PtiO2) < 20 mmHg on any side of the skull.*
  *Near infrared spectroscopy-based cerebral oxygen saturation (SB-CO2) < 50% at any side of the skull or a difference of 20% of SB-CO2 between sides.*
  *Jugular oxygen saturation (SjvO2) or near infrared spectroscopy-based jugular saturation (SB-SjvO2) <55% or >75% at any side of the head/neck.*
  *When neuromonitoring is not available, follow-up Imaging (CT scan) showing the presence of new lesions (mass, edema) or worsening of previous ones (increased volume of lesion or edema).*
g *High-risk phenotypes for NW include patients with baseline conditions associated with poor brain compliance/brain oxygenation reserve (anatomical variants or pre-existing conditions) specified in* Table 1. *These patients will require early invasive/non-invasive neuromonitoring to detect subclinical NW after TBI*,* and do not delay action for managing it before additional tissue damage occurs.*

a *NW is a life-threatening emergency that needs to be identified before irreversible brain tissue damage occurs.*
b *The development and implementation of institutional protocols for early detection and early treatment of NW in centers taking care of TBI patients is required as an indicator of quality of care.*
c *NW can be defined as the deterioration of at least two evaluation parameters (clinical, imaging, or invasive/non-invasive neuromonitoring) that induced an associated medical and/or surgical intervention.*
d *A framework of stratification for NW into 3 phenotypes is proposed to identify and act as soon as possible in TBI patients* (Fig. 1):
  a *Established NW.*
  b *Subclinical NW.*
  c *High Risk Phenotype for NW.*

**Table 1:** Conditions that predispose to a high-risk phenotype

Pre-Existing Anatomical High-Risk Conditions for NW after TBI	Systemic High-Risk Conditions that can predispose to NW after TBI
Cranial stenosis without treatment (trigonocephaly, scaphocephaly, or plagiocephaly)	**1. Associated with cerebral hypoperfusion** Carotid or vertebral atherosclerosis (stenosis, dissection) Chronic vascular diseases are associated with arterial atherosclerotic or venous thrombosis. Cancer Antiphospholipid syndrome Vera Polycythemia Moya-Moya disease
	**2. Conditions that affect cerebral autoregulation** Aging Diabetes microangiopathy Severe arterial hypertension Sepsis Hepatic Failure Hypoxic-anoxic encephalopathy Moya-Moya disease Chronic renal failure
Brain´s vascular agenesis, malformation, hypoplasia, or stenosis (developmental venous anomaly (DVA), venous malformation (VM), dural arteriovenous fistula (DAVF), dural venous sinus agenesis/hypoplasia, arterial duplication, accessory arterial vessels, or abnormal origin of arteries).	**3. CSF dynamics compromise** Idiopathic Intracranial Hypertension Chronic use of isotretinoin or tetracycline Systemic Lupus Erythematosus, Sarcoidosis Multiple Sclerosis Previous Stroke, SAH, TBI, meningitis Brain or spinal tumors
Congenital or acquired cranial hyperostosis (abnormal thickening with overgrowth of skull bones on the inner surface >2 cm).	**4. Cerebral venous flow alteration** Jugular venous thrombosis Intrathoracic pressure increases (pneumothorax, ARDS, hemothorax, mechanical ventilation) Intrabdominal pressure increase (obesity, ileus, hemo or pneumoperitoneum)
Congenital or acquired anatomical obstruction of the cerebrospinal fluid flow at the ventricles or cisterns (obstructive hydrocephalus, Chiari malformation, or syringomyelia).	**5. Poor brain oxygenation reserve** Obstructive sleep apnea Chronic Obstructive Pulmonary Disease Severe Asthma Pulmonary fibrosis Heart Failure Chronic Anemia Carbon monoxide poisoning
Intracranial or extracranial tumors that generate anatomical skull vault/base distortion by physically pushing, stretching, or infiltrating surrounding brain tissue.	**6. Drugs** Vasodilators (increase cerebral blood volume) Anesthetic agents Noradrenaline at high doses Anticoagulants/antithrombotic (increase bleeding risk)
Previous anatomical distortion or brain tissue disruption associated with TBI, stroke, or previous cranial surgery, including Decompressive craniectomy.	**7. Others** Epilepsy Cerebral fat embolism Coagulation disorders (hemophilia, coagulation factor deficit, hepatic diseases, chronic kidney disease/renal failure) Conditions associated with secondary insults: Hypoglycemia- Hyperglycemia (Diabetes mellitus) Hyponatremia (adrenal insufficiency)

## Discussion

Neurological deterioration after TBI is a critical situation that requires early identification and management [1–6]. Clinical studies demonstrate that NW increases mortality and induces the worst functional outcomes [1, 2]. Recent data from the TRACK-TBI study confirm that NW in the emergency department is associated with higher requirements of invasive monitoring, neurosurgical intervention, ICU admission, and obviously with poorer outcomes [3]. This problem is more pronounced in low- and middle-income countries, where decreased access to neuromonitoring, timely surgery, and neurocritical care may delay recognition and treatment, exacerbating worst outcomes [14–18]. The research on NW remains limited in both quantity and quality [4]. A major contributor to this knowledge gap is the heterogeneity of NW definitions [4, 18]. Commonly, NW has been identified exclusively by clinical examination and imaging parameters, and only a few definitions incorporate neuromonitoring variables [4]. The lack of a unified and stratified framework decreases comparability across studies and induces barriers for the development of standardized management strategies. Definitions based on clinical findings (typically low GCS, loss of pupillary reactivity, or development of new focal deficits) are limited by interobserver variability and confounding factors (e.g., sedation, neuromuscular paralysis) [1–3, 6, 18–24]. This clinical deterioration may only become noticeable once brain tissue herniation is present or irreversible [1–3, 6, 18–24]. Imaging may also be insufficient to capture evolving secondary injuries because associated patterns of brain herniation are a late sign to avoid secondary injuries in the tissue [4, 6, 25–31].

In the current era of neurotraumatology care, neuromonitoring, both invasive and non-invasive, plays a critical role in the early detection of various causes of NW [32–41]. Electroencephalography is a useful tool to detect and treat seizures or non-convulsive states in deeply sedated patients; however, sedative drugs (especially benzodiazepines, propofol, barbiturates) have a pronounced slowing effect on EEG activity. Thus, the appearance of ‘spindles’ (with light sedation) or a burst-suppression pattern (with deep sedation) creates significant difficulties in the differential diagnosis between drug-induced encephalopathy and ischemic/epileptic changes. Furthermore, in paralyzed individuals, the EEG can register muscle artifacts. Other environmental artifacts interfere with obtaining an adequate EEG recording. Given its complexity, regardless of the modality used (qualitative or quantitative), interpretation in the ICU requires the assistance of a specialist [42–44]. Changes in intracranial pressure wave morphology (a marker of cerebral compliance), cerebral perfusion, or brain oxygenation should lead to early consideration of treatment escalation [19, 45], particularly in patients who cannot be reliably examined at the bedside. Multimodal neuromonitoring becomes necessary to detect early deterioration [16, 41]. Portable non-invasive neuromonitoring technologies are increasing the possibilities for earlier identification of NW, even in prehospital settings [32–41]. Although definitive evidence is still emerging, the consistency of physiological signals across modalities supports their inclusion of these systems, in a unified diagnostic framework for NW.

Clinical studies have not definitively assessed whether alterations in physiological parameters obtained from systemic and or neuromonitoring parameters precede clinical deterioration, nor whether they are sufficient on their own to define NW. Some studies suggest that alteration in classical vital signs monitoring can precede NW, but these conclusions remain to be validated [46, 47]. Nevertheless, evidence-based medicine is outdated in the face of the current rapid growth of knowledge, especially related to novelty monitoring technologies like the ones discussed in the Brussels Consensus for Non-Invasive ICP Monitoring [32]. This proposed new definition and stratified framework provides an innovative and coherent foundation for moving from a reactive to a proactive approach to the identification and management of NW after TBI. The consistency of physiological signals across modalities strongly supports their inclusion within a unified conceptual framework.

An important point to highlight is that our proposal of definitions can also help to standardize future research addressing the mechanisms of NW as well as strategies for its prevention and treatment (Table 2). These definitions attempt to reflect an innovative proposal, a paradigm shift towards an integrated and personalized view with the objective of early detection and preventing the lethal consequences of NW, beyond the current evidence quality, which, obviously, until now, is not the best, considering these new definitions. In the definitions associated with high-risk phenotypes, we consider identifying clinical conditions that have some predisposition for developing NW. In this context, we can identify two main groups **(**Table 1)**.** The first group is characterized by the presence of structural or anatomical abnormalities of the cranial cavity, brain parenchyma, cerebral vasculature (arterial or venous), or cerebrospinal fluid (CSF) physiology that, in one way or another, affects cerebral compliance in a pre-injury state [48–55]. The second group consists of systemic conditions that, in various ways, facilitate the impairment of intracranial and brain dynamics (hypoxemia, hypotension, hyponatremia, hypoglycemia, etc.), hemorrhages, inflammation, or alter key cerebral physiological phenomena such as perfusion, autoregulation, or oxygenation [4, 56–58]. We recommend that all patients with high-risk phenotypes and TBI need to be monitored with serial non-invasive techniques or continuous invasive techniques to define as early as possible, a subclinical NW stage (Table 3). Once a patient with TBI is in a subclinical NW stage (with the presence of concomitant pathophysiological changes in two or more invasive or non-invasive neuromonitoring modalities), early action to improve the problems needs to be taken.

**Table 2:** Paradigm shifts proposed to modernize the definition, detection, and management of NW in TBI

Traditional Approach	Modern Paradigm Shift	Rationale & Implications
1. Reactive care — NW recognized only after overt clinical deterioration.	**Proactive early detection** based on physiological trends and multimodal monitoring.	Clinical signs emerge late; early detection of physiological changes allows for timely action before irreversible damage has occurred.
2. Clinical-only definitions relying predominantly on the neurological exam.	**Multimodal integration** (clinical, radiological, invasive, and non-invasive monitoring).	Enhances sensitivity, especially in sedated/comatose patients; aligns with precision medicine.
3. Incidental or provider-dependent recognition	**Structured institutional surveillance and standardized protocols**	Reduces missed deterioration; improves reproducibility and timely clinical decision-making.
4. Heterogeneous and unstratified definitions	**Unified**,** stratified framework**: pre-NW (high-risk phenotype), subclinical NW, established NW.	Enables consistent research methodology, clearer diagnostic triggers, and algorithmic management.

**Table 3:** Suggested pragmatic management principles according to the NW phenotype

NW Phenotype	Suggested Management Approach	Rationale
High-risk phenotype (Pre-NW)	• Close clinical surveillance, preferably in an ED or ICU setting. • Scheduled repeat CT within 6–12 h after the initial abnormal scan. • Serial non-invasive monitoring since the ED (e.g., ONSD, TCD PI, waveform compliance, NPI). • In the presence of “borderline” surgical lesions, consider lowering the operative threshold when two non-invasive modalities are already abnormal.	Patients who present with at least 2 abnormal neuromonitoring parameters at baseline have subclinical NW.
Subclinical NW	• Early optimization of systemic physiology (e.g., oxygenation, ventilation, MAP, temperature). • Repeat CT earlier than scheduled (e.g., when new physiological changes arise) to detect radiological progression. • Full correction of systemic derangements and escalation of monitoring. • Consider escalation to invasive monitoring in subclinical NW or even another more aggressive surgical approach.	Worsening in invasive or non-invasive neuromonitoring parameters may identify early secondary neurological insults before clinical changes.
Established NW	• Immediate clinical reassessment and stabilization (within minutes). • Urgent repeat CT if not already performed. • Prompt surgical intervention when indicated, ideally within one hour of identification.	Documented deterioration from baseline probably requires urgent surgical intervention to prevent irreversible secondary injury.

ICU: Intensive care unit; CT: computed tomography; ONSD: optic nerve sheath diameter; TCD PI: pulsatility index evaluated through transcranial Doppler; MAP: Mean arterial pressure; GCS: Glasgow coma scale; NPI: Neurologic pupillary index

In low-resource settings or in areas where neuromonitoring is not available (either invasive or non-invasive), serial imaging with changes in the primary injury pattern (increasing lesion) (even in the absence of clinical deterioration) can be considered for subclinical NW. In this case, it’s essential to manage all cerebral or systemic physiological disarrangements and to request either non-invasive or invasive neuromonitoring, or to consider a more aggressive surgical approach.

Finally, what we aim to prevent (the established NW) will be characterized by the classic clinical or imaging signs of brain herniation [1, 2, 10–12]. In this case, medical or surgical management does not differ from the classic protocols of emergency aggressive therapy [1, 2, 10–12].

### Limitations of the consensus

This consensus process has several limitations that should be acknowledged. First, the sample size of 20 respondents, while appropriate for a Delphi consensus process, is relatively small and may not capture the full diversity of opinion across all Latin American countries and practice settings. Second, the dominance of neurosurgeons (55%) and critical care physicians (40%) may introduce specialty-specific perspectives, though this reflects the primary specialties involved in TBI care. We recognize that the development of complex clinical constructs through expert consensus is inherently subject to several well-described methodological and cognitive challenges, including conceptual heterogeneity, variability in expert perspectives, and the risk of implicit bias in consensus formation. In designing our process, we try to mitigate these barriers by predefining a consensus threshold (≥ 80%), including a multidisciplinary panel, and structuring iterative feedback across rounds. Detailed disagreement rates are described in the supplementary material. The proposed definition statements should be considered hypothesis-generating for future research in order to validate the new concept.

### Future research

Future prospective studies will be necessary to externally validate the construct and assess its predictive and clinical utility. The future research needs to be focused on defining what combination of risk factors may best predict the development of NW, and the role of serum biomarkers for predicting NW [59–61]. There is an urgent need for improving the knowledge about non-invasive thresholds for defining NW and the minimum requirements for NW surveillance in low-resource settings. It will also be important to explore the role of AI and machine learning models to identify endotypes that predispose TBI patients to have a higher risk for NW [40, 62].

## Conclusions

After TBI, NW is a high-risk situation associated with worse outcomes. The traditional diagnosis system based on clinical and imaging changes associated with an active brain herniation process seems to reflect a very late stage for timely management to avoid brain tissue damage. A new stratified framework integrating definitions for patients with high-risk phenotypes, Subclinical NW, and Established NW offers a more coherent and proactive approach focused on early recognition and management of this complication. This paradigm shift moves from reactive to anticipatory diagnosis and management to improve functional outcomes and decrease mortality in TBI patients. Future research should consistently apply to validate this integrated definition across diverse healthcare settings. Such efforts are essential to identify populations at risk, refine predictive models, and develop effective protocols for early recognition and treatment of NW worldwide.

## Supplementary Information

Below is the link to the electronic supplementary material.

Supplementary Material 1

## Data Availability

No datasets were generated or analysed during the current study.
