# Supplementary material for: Neuroworsening in traumatic brain injury: A consensus of the Latin American Brain Injury Consortium (LABIC) and the Latin American Federation of Neurosurgical Societies (FLANC) expert group
Source: Neurosurg Rev. 2026 May 7;49(1):396. doi: 10.1007/s10143-026-04284-z (PMC13152910; doi:10.1007/s10143-026-04284-z)
Supplement: Supplementary file 1 — Supplementary Material 1 [file 10143_2026_4284_MOESM1_ESM.pdf]

Supplementary Material

Search Strategy:

Filters: Clinical Conference, Clinical Trial, Clinical Trial, Phase IV, Guideline, Meta-Analysis, Multicenter Study, Practice Guideline, Pragmatic Clinical Trial, Randomized Controlled Trial, Scoping Review, Systematic Review, Humans, from 2010 - 2026

(((("brain injuries, traumatic"[MeSH Terms] OR ("brain"[All Fields] AND "injuries"[All Fields] AND "traumatic"[All Fields]) OR "traumatic brain injuries"[All Fields] OR ("traumatic"[All Fields] AND "brain"[All Fields] AND "injury"[All Fields]) OR "traumatic brain injury"[All Fields]) AND ("diagnosable"[All Fields] OR "diagnosis"[All Fields] OR "diagnosis"[MeSH Terms] OR "diagnosis"[All Fields] OR "diagnose"[All Fields] OR "diagnosed"[All Fields] OR "diagnoses"[All Fields] OR "diagnosing"[All Fields] OR "diagnosis"[MeSH Subheading])) OR ("manage"[All Fields] OR "managed"[All Fields] OR "management s"[All Fields] OR "managements"[All Fields] OR "manager"[All Fields] OR "manager s"[All Fields] OR "managers"[All Fields] OR "manages"[All Fields] OR "managing"[All Fields] OR "management"[All Fields] OR "organization and administration"[MeSH Terms] OR ("organization"[All Fields] AND "administration"[All Fields]) OR "organization and administration"[All Fields] OR "management"[All Fields] OR "disease management"[MeSH Terms] OR ("disease"[All Fields] AND "management"[All Fields]) OR "disease management"[All Fields])) AND ("deteriorate"[All Fields] OR "deteriorated"[All Fields] OR "deteriorates"[All Fields] OR "deteriorating"[All Fields] OR "deterioration"[All Fields] OR "deteriorations"[All Fields] OR "deteriorative"[All Fields] OR ("worsen"[All Fields] OR "worsened"[All Fields] OR "worsening"[All Fields] OR "worsening"[All Fields] OR "worsens"[All Fields]) OR ("impair"[All Fields] OR "impaired"[All Fields] OR "impairment"[All Fields] OR "impairments"[All Fields] OR "impairing"[All Fields] OR "impairment"[All Fields] OR "impairments"[All Fields] OR "impairs"[All Fields])) AND ("guideline"[Publication Type] OR "guidelines as topic"[MeSH Terms] OR "guidelines"[All Fields] OR ("consensual"[All Fields] OR "consensually"[All Fields] OR "consensus"[MeSH Terms] OR "consensus"[All Fields]) OR ("protocol"[All Fields] OR "protocol s"[All Fields] OR "protocolized"[All Fields] OR "protocols"[All Fields]))) AND ((clinical conference[Filter] OR clinical trial[Filter] OR clinical trial phase iv[Filter] OR guideline[Filter] OR meta-analysis[Filter] OR multicenter study[Filter] OR practice guideline[Filter] OR pragmatic clinical trial[Filter] OR randomized controlled trial[Filter] OR scoping review[Filter] OR systematic review[Filter]) AND (humans[Filter]) AND (2010:2026[pdat]))

DELPHI RESULTS

Expert Panel Demographics

The members of the Latin American Federation of Neurosurgical Societies (FLANC) and the members of the Latin American Brain Injury Consortium (LABIC), were selected base in specific inclusion criteria: (a) > 10 years' experience in the management of TBI; (b) active involvement in acute care management of TBI population; (c) representation of pertinent disciplines (neurosurgeons, neurocritical care and general intensivists, neurologists and anesthesiologists); (d) geographic diversity around Latin America (Central, South and Caribbean region); (e) training in systematic searches; and (f) ability to commit time to the statement development process. The working group was divided into two subgroups: one in charge of the design of the exercise, planning it, organizing the logistics and the developing the first document draft (methodological group) who did not participate in the voting process (n=4) and the group of panelists (inclusion criteria; n=20) in charge of the statement developing and voting with active participation in the discussions (expert group). Prior to the first Delphi round, the results of the systematic search were distributed to all consensus members. The methodological group summarized the evidence and shared it with each member of the voting group. The first round of the Delphi consensus process achieved a 95.2% response rate, with 20 of 21 invited experts completing the survey. The expert panel demonstrated strong multidisciplinary representation, with neurosurgeons comprising 55.0% (n 11) of respondents, critical care medicine specialists including anesthesiologists 40.0% (n 8), and neurologists 5.0% (n 1) ***(Figure 1, Table 1).*** Regarding practice settings, respondents represented diverse healthcare environments across Latin America. The largest proportion practiced in academic medical centers or university hospitals (35.0%, n 7), followed by public hospital/healthcare systems (25.0%, n 5). Mixed practice settings were also well-represented, with 15.0% (n 3) working in mixed academic-private environments and 15.0% (n 3) in mixed public-private settings. Private hospital/healthcare systems accounted for 10.0% (in 2) of respondents ***(Figure 1, Table 1).***

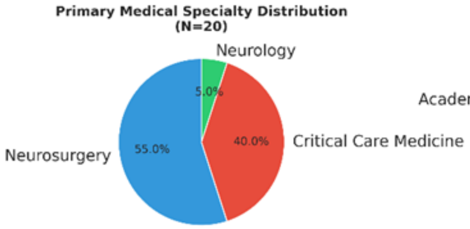

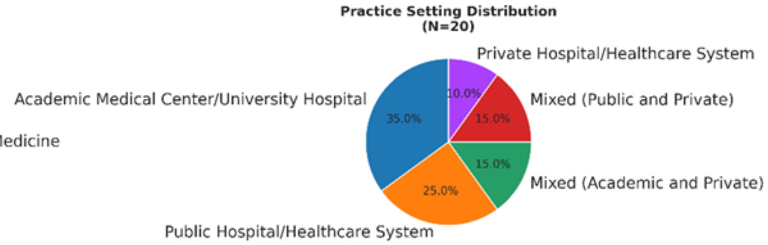

Category	Item	Count	Percentage
Specialty	Neurosurgery	11	55%
Specialty	Critical Care Medicine	8	40%

Specialty	Neurology	1	50%
Practice Setting	Academic Medical Center/University Hospital	7	35%
Practice Setting	Public Hospital/Healthcare System	5	25%
Practice Setting	Mixed (Academic and Private)	3	15%
Practice Setting	Mixed (Public and Private)	3	15%
Practice Setting	Private Hospital/Healthcare System	2	100%

Consensus Performance

The first Delphi round demonstrated exceptional consensus across all proposed statements, with 100% of statements (27/27) achieving the predefined consensus threshold of ≥80% agreement **(Table 2, Table 3, Figure 2, Figure 3, Figure 4).** This remarkable level of agreement suggests strong foundational consensus among Latin American neurotrauma experts regarding the core concepts, definitions, and criteria for neuroworsening in TBI. Core Statements (A-D): All four core statements achieved unanimous or near-unanimous consensus.

**Category**	**Total Statements**	**Consensus Achieved**	**Consensus Rate**
Core Statement (A-D)	4	4	100%
Established NW Criteria (E, E.1-E.5)	6	6	100%
Subclinical NW Criteria (F, F.1-F.15)	16	16	100%
High-Risk Phenotype (G)	1	1	100%
TOTAL	27	27	100%

**Statement ID**	**Agree (n)**	**Total (N)**	**Agreement (%)**	**Consensus (≥80%)**
A	20	20	100%	Yes
B	19	20	95%	Yes
C	20	20	100%	Yes

D	20	20	100%	Yes
E	20	20	100%	Yes
E.1	19	20	95%	Yes
E.2	19	20	95%	Yes
E.3	19	20	95%	Yes
E.4	19	20	95%	Yes
E.5	19	20	95%	Yes
F	20	20	100%	Yes
F.1	18	20	90%	Yes
F.2	19	20	95%	Yes
F.3	19	20	95%	Yes
F.4	19	20	95%	Yes
F.5	19	20	95%	Yes
F.6	19	20	95%	Yes
F.7	19	20	95%	Yes
F.8	20	20	100%	Yes
F.9	19	20	95%	Yes
F.10	19	20	95%	Yes
F.11	20	20	100%	Yes
F.12	20	20	100%	Yes
F.13	19	20	95%	Yes
F.14	19	20	95%	Yes
F.15	19	20	95%	Yes
G	20	20	100%	Yes

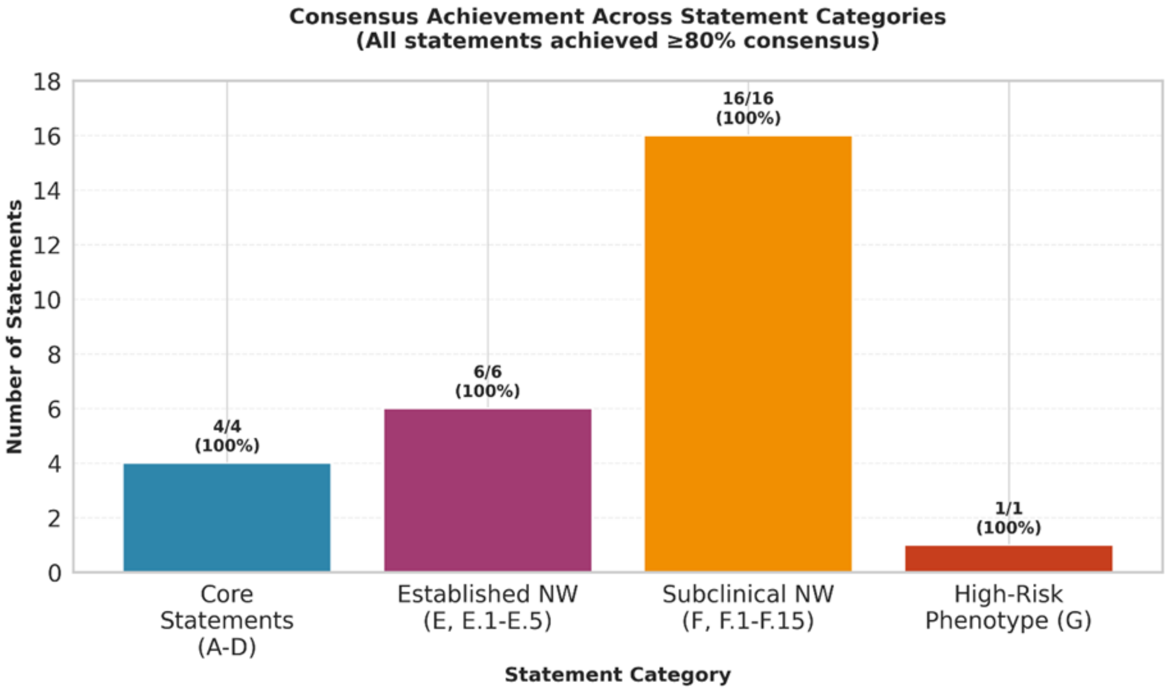

Consensus Achievement Across Statement Categories
(All statements achieved ≥80% consensus)
Number of Statements
0
2
4
6
8
10
12
14
16
18
4/4 (100%)
6/6 (100%)
16/16 (100%)
1/1 (100%)
Core Statements (A-D)
Established NW (E, E.1-E.5)
Subclinical NW (F, F.1-F.15)
High-Risk Phenotype (G)
Statement Category

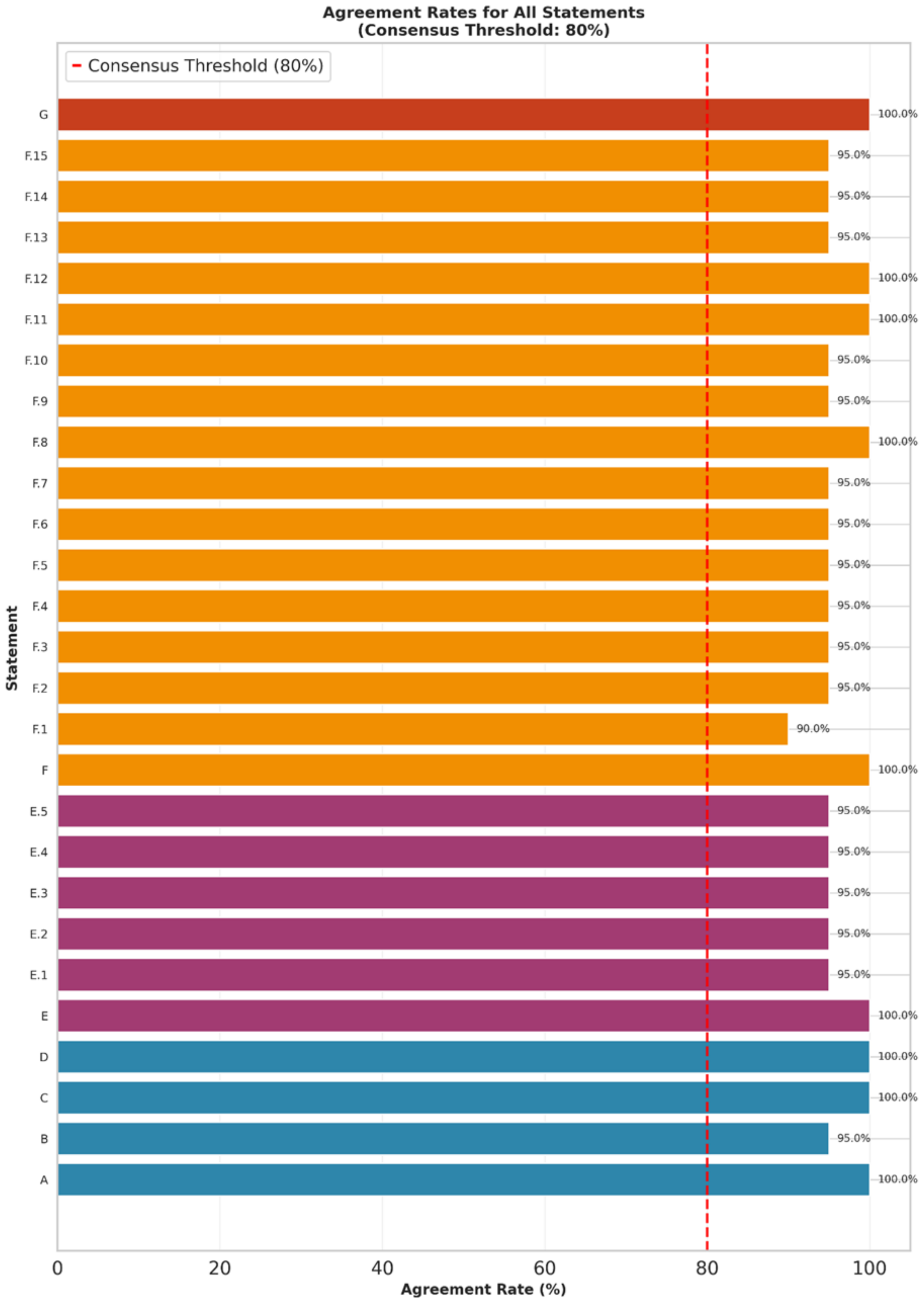

Agreement Rates for All Statements
(Consensus Threshold: 80%)
Consensus Threshold (80%)
G
F.15
F.14
F.13
F.12
F.11
F.10
F.9
F.8
F.7
F.6
F.5
F.4
F.3
F.2
F.1
F
E.5
E.4
E.3
E.2
E.1
E
D
C
B
A
100.0%
95.0%
95.0%
95.0%
100.0%
100.0%
95.0%
95.0%
100.0%
95.0%
95.0%
95.0%
95.0%
95.0%
95.0%
90.0%
100.0%
95.0%
95.0%
95.0%
95.0%
95.0%
100.0%
100.0%
100.0%
95.0%
100.0%
Statement
0
20
40
60
80
100
Agreement Rate (%)

The distribution of agreement rates across all 27 statements demonstrated remarkable consistency ***(Figure 4).*** Three distinct agreement levels were observed: 100% agreement (n 11 statements, 40.7%), 95% agreement (on 15 statements, 55.6%), and 90% agreement (n 1 statement, 3.7%). Notably, no statement fell below the 80% consensus threshold, and the lowest agreement rate observed was 90%, substantially exceeding the predefined consensus threshold. This distribution pattern suggests that the proposed neuroworsening framework, definitions, and criteria are well-aligned with current expert opinion and clinical practice across Latin American neurotrauma centers.

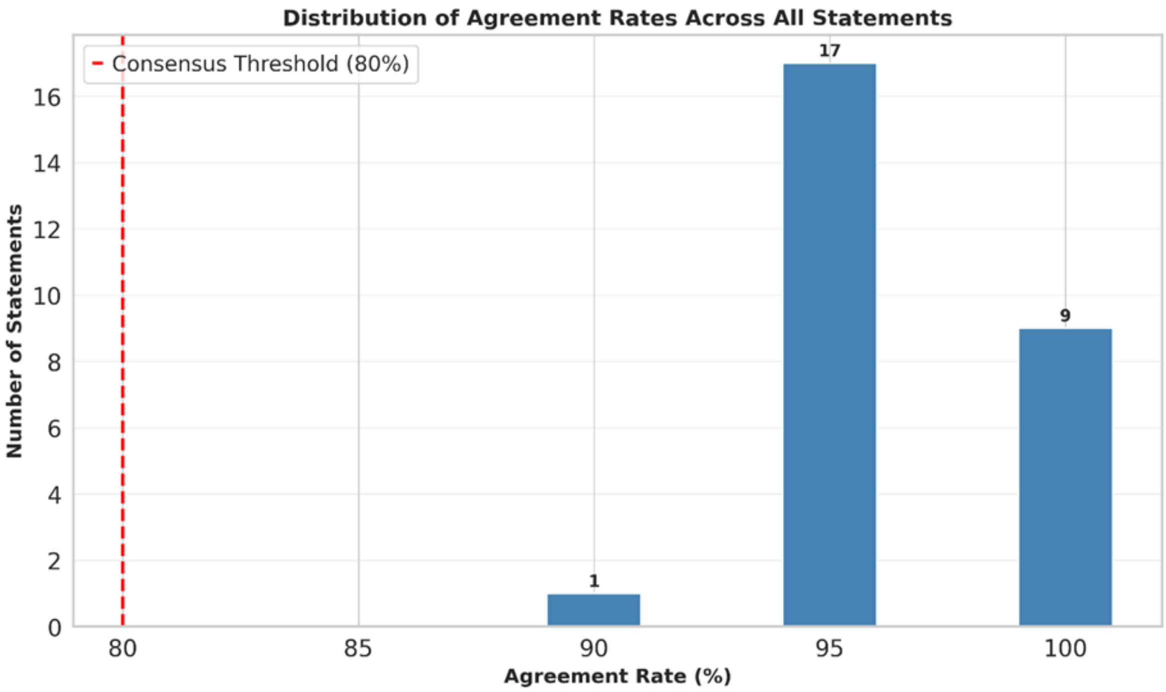

Established Neuroworsening Criteria (E, E.1-E.5)

All six statements regarding established neuroworsening criteria achieved consensus ranging from 95.0% to 100%. Statement E, the overarching definition of "Established NW as defined by traditional criteria (clinical and imaging findings associated with brain herniation)," achieved 100% agreement (20/20 respondents). The five specific criteria for Established NW each achieved 95.0% agreement (19/20 respondents):

- E.1: Glasgow Coma Score (GCS) reduction of at least 2 points in motor component

- E.2: Anisocoria of at least 1 mm of difference

- E.3: Abnormal motor response (unilaterally or bilaterally)

- E.4: Midline shift on computed tomography (CT) imaging

- E.5: Basal cistern compression (unilaterally or bilaterally) on CT imaging

The consistent 95% agreement across all individual Established NW criteria demonstrates strong consensus on traditional clinical and radiological markers of neuroworsening. However, qualitative comments revealed some concerns regarding specific thresholds, particularly the 2-point GCS motor component reduction, with some experts suggesting that a 1-point reduction might be more clinically relevant for early detection.

Subclinical Neuroworsening Criteria (F, F.1-F.15)

All sixteen statements regarding subclinical neuroworsening criteria achieved consensus, with agreement rates ranging from 90.0% to 100%. Statement F, the overarching definition of "Subclinical NW as the deterioration of at least two parameters in neuromonitoring (invasive or non-invasive), inducing medical and/or surgical interventions," achieved 100% agreement (20/20 respondents). Among the specific neuromonitoring criteria, four achieved 100% agreement:

- F.8: ICP >22mmHg (20/20 respondents)

- F.11: Sharp waves, spikes, or spike-and-wave complexes or increased theta: alpha ratio or theta: beta ratio during EEG monitoring (20/20 respondents)

- F.12: Brain oxygen tissue pressure (PtiO2) <20 mmHg (20/20 respondents)

Eleven criteria achieved 95.0% agreement (19/20 respondents):

- F.2: Neurologic pupillary index (NPi) <3

- F.3: Quantitative pupillary index (QPi) <3

- F.4: Optic nerve sheath diameter (ONSD) >6mm on any side

- F.5: Peak 2 (P2) > Peak 1 (P1) pattern during ICP waveform analysis

- F.6: P2/P1 ratio >1.4 on non-invasive ICP monitoring

- F.7: Normalized time to peak (nTTP) >0.3 during non-invasive ICP monitoring

- F.9: Medial cerebral artery (MCA) diastolic flow velocity <20cm/sec on transcranial Doppler

- F.10: Pulsatility index (PI) >1.3 during TCD monitoring

- F.13: Near infrared spectroscopy-based cerebral oxygen saturation (SB-CO2) <50% or difference of 20% between sides

- F.14: Jugular oxygen saturation (SjvO2) or SB-SjvO2 <55% or >75%

- F.15: New lesions, increased volume of existing lesions, or increased edema on imaging

One criterion achieved the lowest (though still consensus-level) agreement:

- F.1: Pupillometry maximum contraction velocity (MCV) difference >1mm/sec between eyes (90.0%, 18/20 respondents)

The high level of consensus across diverse neuromonitoring modalities reflects the panel's recognition of the importance of multimodal monitoring in detecting subclinical neuroworsening. Qualitative comments highlighted the need for careful wording regarding baseline versus change in parameters, and the importance of distinguishing subclinical neuroworsening from imaging changes that would constitute established neuroworsening.

High-Risk Phenotype Statement (G)

Statement G, defining "High-risk phenotypes for NW as patients with baseline conditions associated with poor brain compliance/brain oxygenation reserve requiring early invasive/non-invasive neuromonitoring," achieved 100% agreement (20/20 respondents). This unanimous consensus supports the proactive identification and enhanced monitoring of high-risk patients.

Qualitative Comments and Expert Feedback

Twenty-two qualitative comments were provided across various statement categories. Key themes emerging from the comments included:

1. Threshold refinement: Several experts suggested that the 2-point GCS motor component reduction threshold for Established NW might be too restrictive, recommending consideration of a 1-point reduction for earlier detection.

2. Parameter wording: Experts emphasized the importance of distinguishing between absolute values and changes from baseline, particularly for neuromonitoring parameters.

3. Imaging criteria clarification: Comments suggested specifying "new onset" or "worsening" for criteria such as anisocoria and midline shift to better capture deterioration rather than baseline abnormalities.

4. ONSD monitoring: One expert noted that ONSD should be evaluated as a serial parameter rather than relying on a single absolute value.

5. Integration with clinical assessment: Multiple comments emphasized that neuromonitoring criteria for subclinical NW should be interpreted in the context of clinical examination, particularly GCS motor component.

6. Definition of high-risk phenotype: Experts provided detailed suggestions for expanding the definition of high-risk phenotypes to include specific clinical scenarios such as moderate TBI and patients with already abnormal monitoring parameters at initial evaluation.

Principal Aspects of the Consensus

This first round of the Delphi consensus process yielded exceptional results, with all 27 proposed statements achieving the predefined consensus threshold of ≥80% agreement among Latin American neurotrauma experts. The unanimous or near-unanimous consensus across core statements, established neuroworsening criteria, subclinical neuroworsening criteria, and high-risk phenotype definitions demonstrate strong alignment between the proposed framework and current expert opinion in the region. The 100% consensus achievement rate in the first round is remarkable and suggests that the proposed neuroworsening framework addresses a critical gap in TBI management with concepts that resonate strongly with clinical practice. This high level of agreement may reflect the urgent clinical need for standardized definitions and criteria for neuroworsening, as well as the careful development of statements based on existing literature and clinical experience.

Interpretation of Core Statements in the Consensus

The universal or near-universal agreement on core statements (A-D) provides strong validation for the fundamental concepts underlying the neuroworsening framework. The 100% agreement on Statement A confirms that experts uniformly recognize neuroworsening as a life-threatening emergency requiring timely identification. This consensus is particularly important as it establishes clinical urgency and priority that should be assigned to neuroworsening detection and management protocols. The 95% agreement on Statement B, regarding institutional protocols as quality indicators, reflects broad recognition that systematic approaches to neuroworsening are essential for optimal TBI care. The single dissenting response may reflect practical concerns about implementation challenges in resource-limited settings, though the qualitative comment was supportive of the concept. This near-unanimous support provides strong justification for incorporating neuroworsening protocols into quality improvement initiatives and institutional guidelines. The 100% consensus on Statement C validates the multi-parametric definition of neuroworsening, requiring deterioration in at least two evaluation parameters. This approach balances sensitivity and specificity by reducing false positives that might occur with single-parameter changes while maintaining adequate sensitivity for detecting true clinical deterioration. However, qualitative feedback suggested refinement of the wording to clarify whether this applies to all neuroworsening or specifically to subclinical neuroworsening, indicating a need for clearer delineation in subsequent rounds. The unanimous support for Statement D confirms expert acceptance of the three-phenotype classification system (Established NW, Subclinical NW, High-Risk Phenotype). This stratification framework provides a structured approach to risk assessment and monitoring intensity, potentially enabling more targeted resource allocation and earlier intervention.

Established Neuroworsening Criteria: Strong Consensus with Refinement Opportunities

The 95-100% consensus across all established neuroworsening criteria (E, E.1-E.5) demonstrates strong agreement on traditional clinical and radiological markers of deterioration. The 100% agreement on the overarching definition (Statement E) confirms that the concept of established neuroworsening, based on clinical and imaging findings associated with brain herniation, is well-accepted. However, qualitative feedback revealed important nuances regarding specific thresholds. Some experts questioned whether the 2-point GCS

motor component reduction threshold might be too restrictive, potentially delaying identification of clinically significant deterioration. Suggestions for a 1-point reduction threshold merit serious consideration in subsequent rounds, as earlier detection could enable more timely intervention. This feedback highlights the tension between specificity (avoiding false positives) and sensitivity (ensuring early detection) that must be carefully balanced in any clinical criteria. The consistent 95% agreement on anisocoria (E.2), abnormal motor response (E.3), midline shift (E.4), and basal cistern compression (E.5) validates these traditional markers. However, expert comments emphasized the importance of specifying "new onset" or "worsening" for these criteria to distinguish acute deterioration from baseline abnormalities present on admission. This refinement would strengthen the criteria by focusing on change rather than absolute findings.

Subclinical Neuroworsening: Comprehensive Consensus Across Monitoring Modalities

The achievement of 90-100% consensus across all 16 subclinical neuroworsening criteria (F, F.1-F.15) represents a major accomplishment, given the diverse neuromonitoring technologies and parameters included. This broad consensus reflects growing recognition of the value of multimodal monitoring in TBI and the importance of detecting deterioration before it becomes clinically apparent. The 100% agreement on the overarching definition (Statement F) confirms acceptance of the concept that subclinical neuroworsening can be identified through neuromonitoring parameter deterioration. The requirement for at least two abnormal parameters before triggering intervention is designed to reduce false positives and unnecessary interventions, though qualitative feedback suggested this should be clarified to distinguish it from imaging changes that would constitute established neuroworsening. Among specific monitoring parameters, the 100% consensus on ICP >22mmHg (F.8), EEG changes (F.11), and PtiO2 <20mmHg (F.12) reflect well-established thresholds with strong evidence bases. These parameters have been extensively studied and are commonly used in neurocritical care, which likely contributed to universal agreement. The 95% consensus on most other parameters (F.2-F.7, F.9-F.10, F.13-F.15) demonstrates strong support for incorporating diverse monitoring modalities, including pupillometry, ultrasound, transcranial Doppler, and near-infrared spectroscopy. This broad agreement suggests that experts recognize the complementary value of different monitoring technologies in providing a comprehensive assessment of brain physiology. The lowest consensus was observed for pupillometry MCV difference (F.1) at 90%, though this still substantially exceeds the consensus threshold. This slightly lower agreement may reflect less familiarity with this specific parameter or questions about the optimal threshold. The qualitative feedback emphasized that parameters like ONSD and pupillometry indices should be evaluated as trends rather than single absolute values, which is an important consideration for implementation. Some experts noted that some subclinical NW parameters may not be routinely measured in all centers, particularly in resource-limited settings.

High-Risk Phenotype: Universal Support for Proactive Monitoring

The 100% agreement on the high-risk phenotype statement (G) confirms strong support for proactive identification of patients who may benefit from enhanced monitoring even before deterioration occurs. This preventive approach aligns with modern precision medicine

concepts and risk stratification strategies. Qualitative feedback provided valuable suggestions for expanding and clarifying the high-risk phenotype definition. One expert proposed a detailed definition including patients with moderate TBI, those with already abnormal monitoring parameters at initial evaluation despite preserved mental status, and those without current evidence of established or subclinical neuroworsening but with concerning baseline features. These suggestions merit incorporation into refined definitions in subsequent rounds. The unanimous support for enhanced monitoring of high-risk patients has important implications for resource allocation and monitoring protocols. It suggests that experts believe the benefits of early detection in high-risk populations justify the costs and resources required for more intensive monitoring.

Strengths and Limitations

This consensus process has several strengths, including the intentional selection of multidisciplinary experts representing diverse practice settings across Latin America, the use of a structured Delphi methodology, and the achievement of a high response rate (95.2%). The exceptional consensus in the first round suggests the proposed framework is well-aligned with clinical practice and expert opinion. However, several limitations should be acknowledged. First, the sample size of 20 respondents, while appropriate for a Delphi consensus process, is relatively small and may not capture the full diversity of opinion across all Latin American countries and practice settings. Second, the dominance of neurosurgeons (55%) and critical care physicians (40%) may introduce specialty-specific perspectives, though this reflects the primary specialties involved in TBI care. Third, the high consensus rates in the first round, while encouraging, may also suggest that some statements were not sufficiently controversial or that the survey design did not adequately capture areas of genuine disagreement.

Clinical Implications

The consensus achieved in this first Delphi round has important implications for TBI care in Latin America and potentially globally. The validated framework provides a foundation for developing standardized protocols for neuroworsening detection, monitoring, and management. The three-phenotype classification system (Established NW, Subclinical NW, High-Risk Phenotype) offers a practical structure for risk stratification and resource allocation. The strong consensus achieved provides confidence that a Latin American position statement on neuroworsening in TBI can be successfully developed through this process, potentially serving as a model for international guideline development in neurotrauma care.
